# Immune landscape and redox imbalance during neurological disorders in COVID-19

**DOI:** 10.1038/s41419-023-06102-6

**Published:** 2023-09-06

**Authors:** Abhimanyu Thakur, Vartika Sharma, Sera Averbek, Lifan Liang, Nirali Pandya, Gaurav Kumar, Alma Cili, Kui Zhang

**Affiliations:** 1 Centre for Regenerative Medicine and Health, Hong Kong Institute of Science and Innovation-CAS Limited, Hong Kong SAR, Hong Kong; 2 Department of Molecular and Human Genetics, Institute of Science, Banaras Hindu University, Varanasi, Uttar Pradesh India; 3 GSI Helmholtzzentrum für Schwerionenforschung GmbH, Darmstadt, Germany; 4 Technische Universität Darmstadt, Darmstadt, Germany; 5 University of Pittsburgh, Pittsburgh, PA USA; 6 Department of Chemistry, Faculty of Sciences, National University of Singapore, Singapore, Singapore; 7 School of Biosciences and Biomedical Engineering, Department of Clinical Research, Galgotias University, Greater Noida, Uttar Pradesh India; 8 Clinic of Hematology, University of Medicine, University Hospital center “Mother Teresa”, Tirane, Albania; 9 State Key Laboratory of Resource Insects, College of Sericulture, Textile and Biomass sciences, Southwest University, Chongqing, China; 10 Cancer Centre, Medical Research Institute, Southwest University, Chongqing, China

**Keywords:** Diseases, Immunopathogenesis

## Abstract

The outbreak of Coronavirus Disease 2019 (COVID-19) has prompted the scientific community to explore potential treatments or vaccines against severe acute respiratory syndrome coronavirus 2 (SARS-CoV-2), the virus that causes the illness. While SARS-CoV-2 is mostly considered a respiratory pathogen, several neurological complications have been reported, raising questions about how it may enter the Central Nervous System (CNS). Receptors such as ACE2, CD147, TMPRSS2, and NRP1 have been identified in brain cells and may be involved in facilitating SARS-CoV-2 entry into the CNS. Moreover, proteins like P2X7 and Panx-1 may contribute to the pathogenesis of COVID-19. Additionally, the role of the immune system in the gravity of COVID-19 has been investigated with respect to both innate and adaptive immune responses caused by SARS-CoV-2 infection, which can lead to a cytokine storm, tissue damage, and neurological manifestations. A redox imbalance has also been linked to the pathogenesis of COVID-19, potentially causing mitochondrial dysfunction, and generating proinflammatory cytokines. This review summarizes different mechanisms of reactive oxygen species and neuro-inflammation that may contribute to the development of severe COVID-19, and recent progress in the study of immunological events and redox imbalance in neurological complications of COVID-19, and the role of bioinformatics in the study of neurological implications of COVID-19.

## Facts

1. COVID-19 can have long-term neurological complications.
2. Patients with COVID-19 have higher levels of free radicals in their brains, which can affect brain cells including neurons and glia.
3. The redox imbalance during COVID-19 can affect brain cells.
4. Immunological response during COVID-19 can affect brain cells.

## Open Questions

1. Does redox imbalance affect the polarity of microglia in brain?
2. How the redox imbalance affect the immune response and vice-versa during COVID-19 infection?
3. Can the redox imbalance be miniaturized in the form of an organ-on-a-chip?
4. Can the integration of machine learning with advanced bioengineering tools like 3D bioprinting, facilitate the identification of novel therapeutic target for COVID-19?

## Introduction

2019 novel coronavirus disease (COVID-19) is an extremely transmissible disease caused by the severe acute respiratory syndrome coronavirus 2 (SARS-CoV-2) infection [1]. The virus is thought to have originated in Wuhan city of China, in December 2019, followed by a massive outbreak causing a global pandemic [2]. In general, coronaviruses encompass a diverse set of viruses capable of infecting different animals, resulting in mild to severe infections in human respiratory systems. Previously, two other coronaviruses, namely, SARS-CoV and Middle East respiratory syndrome coronavirus (MERS-CoV), surfaced in the years 2002 and 2012 respectively, leading to a lethal respiratory diseases in humans [3]. Unfortunately, the present COVID-19 pandemic due to SARS-CoV-2 has devastatingly surpassed both SARS and MERS in terms of the number of infections and deaths, imposing an astonishing threat to the worldwide public health condition [4, 5]. As per current reports, more than 767 million COVID-19 confirmed cases have been registered, with more than 6.9 million deaths (statistics as of 4:01 pm CEST, 5th July 2023, at https://covid19.who.int/). During SARS-CoV-2 infection, the body’s immune system is significantly affected, which can be attributed to either unsuccessful viral clearance, followed by a devastating “cytokine storm”, leading to systemic inflammation and damage of body organs, which may include coagulopathy and/or hemoglobinopathy [6]. The major symptoms of COVID-19 include fever, dry cough, dyspnea, and eventually leading to death owing to severe conditions such as respiratory failure and myocardial damage, shock, or kidney collapse [7]. Beyond the common prevailing symptoms, emerging neurological complications in COVID-19 patients have gained large attention of biomedical researchers, particularly neuroscientists, and medical practitioners [8–11].

The extreme contagious ability of SARS-CoV-2 has been attributed to the differences in various amino acids in Spike 2 (S2) protein compared to the SARS-CoV [12]. Notably, the receptor of SARS-CoV-2; angiotensin-converting enzyme-2 (ACE2), is also expressed in nervous tissue in addition to the respiratory tract [13–15]. Additional surface receptors, CD147, TMPRSS2, and NRP1 have been found to be the receptors facilitating the entry of SARS-CoV-2 into the central nervous system (CNS) [16–18]. For the entry of SARS-CoV-2 into host cells via ACE2, the virus surface spike protein binds to the ACE2 receptor via its receptor-binding domain (RBD) [19]. CD147 facilitates the entry of SARS-CoV-2 into the host cells through endocytosis [16]. The entry of SARS-CoV-2 via TMPRSS2 can be through two different pathways: pH-dependent or independent. In addition, the expression pattern of proteases in host cells was found to be crucial for segregating SARS-CoV-2 into either pathway. Interestingly, SARS-CoV-2 employs a rapid pH-independent pathway to enter and infect cells in the presence of TMPRSS2. Whereas in the absence of TMPRSS2, SARS-CoV-2 depends on a relatively slow acid-actuated late endosomal pathway for infecting the cells. In addition, endosomal acidification is necessary for the priming of endo-lysosomal proteases to facilitate viral fusion through TMPRSS2 [20]. The expression of NRP1 has also been found to be a novel entry mediator for SARS-CoV-2 infection, which is known for binding furin-cleaved substrates, augmenting the infectivity of SARS-CoV-2 through direct binding of the furin-cleaved S1 fragment of the spike protein to NRP1 on the cell surface [18]. Nevertheless, the expression of major receptors (ACE2, CD147, TMPRSS2, NRP1), facilitating the entry of SARS-CoV-2 in host cells in the nervous tissues indicates their potential role in brain invasion [21, 22]. Recently, many other proteins have been found to be associated with COVID-19 neuropathology, e.g., P2X7. Notably, SARS-CoV-2 infection induces the augmentation of extracellular ATP levels, which stimulates the hyperactivation of P2X7 receptors, leading to the stimulation of NLRP3 inflammasome, a major facilitator of neuro-invasion, as found in neurodegenerative and psychiatric disorders [23]. Another receptor, Pannexin-1 (Panx-1), has been found to be critical for COVID-19 pathogenesis. The opening of Panx-1 was found to be dependent on ACE2/furin/endocytosis, inducing inflammation in various pathological conditions including neurodegeneration [24]. Major neurodegenerative diseases include Alzheimer’s disease, Parkinson’s disease, multiple sclerosis, and amyotrophic lateral sclerosis [25–31].

Interestingly, the inability for spontaneous breathing in COVID-19 patients with the need for intensive care further indicates the loss of involuntary control of breathing in the CNS, causing respiratory insufficiency [32–34]. A severe SARS-CoV-2 infection has also been associated with dysregulation of the immune system [35]. Major research showed that immune responses play a crucial role in the severity of pathogenesis of COVID-19 [36–38]. The infection with SARS-CoV-2 can trigger innate and adaptive immune responses. After viral infection, uncontrolled inflammatory innate responses and compromised adaptive immune responses may cause tissue damage locally as well as systemically [39–41]. The outcome of SARS-CoV-2-induced cytokine storm has been reported to be linked to neurological manifestations [42]. Besides the immunological landscape, redox imbalance has also been found to be involved in COVID-19 pathogenesis. Various studies have shown the involvement of redox imbalance in COVID-19, which is also common in many other viral infections [43, 44]. For instance, the level of serum thiols decreases in COVID-19 patients. Moreover, the SARS-CoV-2 induces mitochondrial dysfunction and produces proinflammatory cytokines through the accumulation of superoxide anions (O_2_.^-^), reactive oxygen species (ROS), and reactive nitrogen species (RNS) [45, 46]. A research result using gene set enrichment analyses showed increase in pathways related to oxidants, and biochemical tests revealed a notable rise in free ROS and drops in uric acid levels for COVID-19 patients. Multivariate analyses found that serum levels of VCAM-1 and ICAM-1 were positively associated with one another, and a decrease in the abundance of single electron oxidants was linked with mortality risk in people with coronavirus. Additionally, IL-17c and TSLP levels could predict the need for intensive care in COVID-19 patients [47]. Moreover, mitochondrial dysfunction is a common feature of viral infections and may contribute to immune system dysfunction. ACE2, which cleaves angiotensin II, plays a vital role in maintaining mitochondrial homeostasis. COVID-19 may downregulate ACE2, impacting mitochondrial function and immune function. ACE2 is under-expressed in chronic diseases, which may explain the higher mortality rate in patients with comorbidities. Mitochondrial dysfunction is also found to be independent of ACE2 expression, suggesting it is a significant factor in COVID-19 disease progression [48]. Therefore, understanding redox imbalance as the molecular underpinnings of COVID-19 can aid in the development of novel therapeutic interventions.

In this review, we are discussing the major neurological complications emerging from COVID-19, the participation of the immunological landscape, and the redox imbalance in the neurological implications of COVID-19. Eventually, the application of bioinformatics for deciphering various molecular targets has also been elaborated.

## Neurological complications in COVID-19

**Fig. 1:**
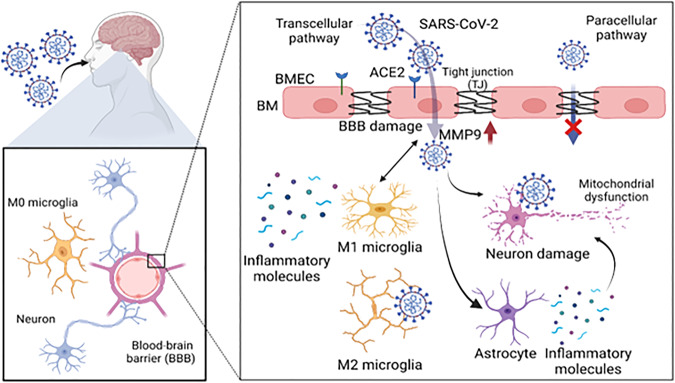
A schematic showing the potential path followed by SARS-CoV-2 to cross the BBB. A typical BBB is composed of various cell types, majorly brain microvascular endothelial cells (BMECs), astrocytes, and pericytes. These cells crosstalk with other brain cells including neurons and microglia. During COVID-19, SARS-CoV-2 crosses the BBB, infecting the BMECs and following the transcellular pathway over the paracellular pathway, where the MMP9-dependent disruption of the basement membrane (BM) occurs. After gaining access to the brain by the SARS-CoV-2, neurons can be infected leading to their damage and mitochondrial dysfunction. This also involves the aggravated inflammatory response such as activation of microglia, and their polarization, followed by the production of inflammatory molecules production, which further promote the BBB damage and injury to neurons (Figure prepared with Biorender).

**Fig. 2:**
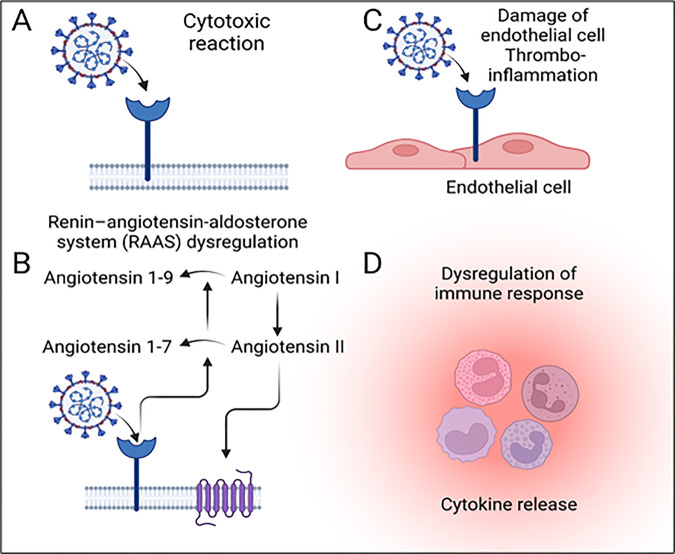
Various mechanisms through which COVID-19 associated pathogenesis takes place after SARS-CoV-2 infection. **A** Cellular damage is mediated directly by the virus. **B** RAAS dysregulation because of ACE2 downregulation associated with the viral entry, leading to reduced cleavage of angiotensin I and angiotensin II. **C** Damage of endothelial cell and thrombo-inflammation; and **D** dysregulated immune response and hyperinflammation elicited by interferon signal inhibition by the virus, depletion of T-cell, and the generation of proinflammatory cytokines, majorly IL-6 and TNF-α (Figure prepared with Biorender).

Owing to the mutational character of SARS-CoV-2, and its ability to enter different organs, SARS-CoV-2 could spread and propagate by various means throughout the body [49]. Interestingly, the entry of SARS-CoV-2 into the brain is quite astonishing due to its ability to cross the blood-brain barrier (BBB), a semi-permeable protective layer around the brain; composed of endothelial cells, astrocytes, and pericytes [50]. Zhang et al. have demonstrated that SARS-CoV-2 can cross BBB through the disruption of the basement membrane without the alteration of the tight junction [51]. In another study by Rhea et al., it was found that the intravenously injected radio-iodinated S1 subunit of its spike protein (I-S1) readily crossed the BBB in mice, was taken up by brain regions, and entered the parenchymal brain space. I-S1 was also taken up by the lung, spleen, kidney, and liver. Importantly, intranasally administered I-S1 also entered the brain, although at levels roughly ten times lower than intravenous administration. Mechanistic studies showed that I-S1 crosses the blood-brain barrier by adsorptive transcytosis and that murine ACE2 is involved in brain and lung uptake, but not in kidney, liver, or spleen [52] (Fig. 1). Subsequently, along with a wide range of symptoms, the appearance of neurological symptoms includes anosmia (partial or full loss of smell), agnosia (inability to interpret sensations), stroke, paralysis, cranial nerve deficits, delirium, encephalopathy, meningitis, and seizures are apparent during the COVID-19 disease progression and its therapy [53, 54]. It is believed that the systemic response against infection is key to several neurological complications in COVID-19. These distinct mechanisms may include systemic dysfunction-induced neurologic injury, renin-angiotensin system (RAS) dysfunction, immune dysfunction, and direct viral invasion of the nervous system, as elaborated and summarized in Table 1 [55–71] and Fig. 2, respectively.

**Table 1:** Proposed mechanisms underlying neuropathogenesis in COVID-19.

Mechanism	Manifestation and indications	Ref.
Systemic dysfunction induced neurological injury	Patients suffering from severe COVID-19 can often experience hypoxemia, which can lead to encephalopathy, a condition of metabolic disruption due to organ failure or medicine side effects. However, those with astrocytic and neuronal injury do not typically present a characteristic pathogenesis when infected with moderate to severe COVID-19. Confusion and changes in consciousness are also common due to high concentrations of circulating proinflammatory cytokines. Brain vessel wall MRI scans of five patients revealed that ventilation therapy for COVID-19-related ARDS affects awakening. The scans showed abnormal contrast enhancement in the vascular wall of the basal skull arteries, possibly indicating the presence of endothelin. Acute hypoxic ischemic damage was observed in the majority of neuropathologic patients infected with COVID-19, along with the presence of hemorrhagic and bland infarcts, microglial activation, and neuronophagia. The other group of patients had symptoms consistent with delayed post-hypoxic leukoencephalopathy, as seen in ARDS, but not related to COVID-19.	[55–60]
RAS dysfunction	The dysregulation of the RAS, due to its reliance on ACE2, may be a factor in the pathophysiology of COVID-19 infection. ACE2 serves to transform angiotensin II into angiotensin-(1-7), which is responsible for vasodilation, antiproliferation, and antifibrotic effects. The binding of SARS-CoV-2 to ACE2, however, can damage vascular endothelial cells by inhibiting mitochondrial and endothelial nitric oxide synthetase activity, leading to secondary cardio- and cerebrovascular effects.	[61–63]
Immune dysfunction	SARS-COV-2 is associated with an irregular systemic immune response including proinflammatory stimulation. *Proinflammatory state:* In cases of severe COVID-19, systemic inflammation has been observed with lasting fever, increased inflammatory markers such as D-dimer and ferritin, and elevated proinflammatory cytokines and markers of inflammation like peripheral tumor necrosis factor (TNF) and interleukin-6 (IL-6). Additionally, thrombophilia, stroke and other thrombotic symptoms may be present due to a proinflammatory state, with complement activation potentially resulting in thrombotic microvascular injury. Cytokines released in the brain can lead to microglial activation and a systemic inflammatory response, resulting in brain injury. There is no established correlation between viral invasion and microglial nodules or neuronophagia; however, microglial activation to phagocytose hypoxic neurons was observed in brain tissue of patients infected with a different virus. *Para-infectious and postinfectious triggers:* In many COVID-19 cases, Guillain-Barré Syndrome appears to be a para-infectious rather than postinfectious complication, not at the start of the infection but in time. One reported case has revealed weakness to be more common than fever and respiratory symptoms. Additionally, there have been reports of a long period of time between the beginning of a viral illness and its accompanying weakness, which is in line with its status as a postinfectious complication.	[56, 64–68]
Viral invasion of the nervous system	SARS-CoV-2 has been found in a majority of brain specimens in post-mortem case series. However, this does not directly correlate with evidence of severe neuropathological conditions, indicating that SARS-CoV-2 may induce a systemic inflammatory response rather than the infection itself causing neural injury. The effects of SARS-CoV-2 on cerebral vessels remain largely unknown. Autopsy reports provide conflicting evidence, pointing to the potential of SARS-CoV-2 to cause direct endothelial invasion, which could be related to endotheliitis in the lung, heart, kidney, liver, and small intestine.	[69–71]

### Effects of COVID-19 on smell and taste

One of the prominent early symptoms of COVID-19 is anosmia (loss of smell) and dysgeusia (taste impairment), which affects most COVID-19 patients [72]. A meta-analysis of 83 studies was performed over 27,000 patients of which 48 percent (95% CI 41.2–54.5) displayed olfactory dysfunction [73]. The initial appearance of COVID-19 might lead to these symptoms without the presence of nasal congestion or discharge, although these are rarely the only clinical manifestations of COVID-19. Patients with COVID-19 showed a disruption of one or both olfactory when examined with MRI screening, showing an abnormal signal. Inflammatory infiltrate and axonal injury in the olfactory tract were found in two autopsy cases, however, it is not known whether this damage was caused by the virus or not. The recovery time of olfactory function can be variable. For instance, 33% of patients in one cohort recovered in 8 days, and in the other cohort of patients from Italy 83% fully recovered in 37 days post the beginning of the symptoms. In addition, 51 patients with anosmia healed in four (84%) to eight months (96%) after the symptom onset. In some cases, anosmia and dysgeusia can last for more than several months and can be accompanied by other viral infections [72, 74–76].

### Encephalopathy

COVID-19 related encephalopathy might also be dependent on other viral infections or factors. Cytokine storm and neurotropism induce an intense inflammatory response, thus altering metabolism or hypoxia, leading to a global dysfunction in the brain. The key symptoms of altered consciousness are delirium, mild confusion, and deep coma. Several case reports confirm the presence of the virus in the cerebrospinal fluid. COVID-19 patients with encephalopathy should be supported with supplemental oxygen therapy and immune modulators, which are supposed to improve the symptoms [53, 77, 78].

### Cardiovascular diseases in COVID-19

COVID-19 related cerebrovascular risk factors are cardiovascular disease, diabetes mellitus, hypertension, smoking, advanced age, and history of stroke. One of the proposed mechanisms of COVID-19-related cerebrovascular risk is that: SARS-CoV-2 binds to ACE2 receptors, resulting in the inactivation of these receptors. This phenomenon promotes the post-ischemic inflammation cascade, which disrupts perfusion in the ischemic zone, and leads to the development of a larger infarct volume in the case of ischemic stroke (IS). Impaired ACE2 function by COVID-19 can also cause hypertensive peaks and impairment of cerebrovascular endothelium. COVID-19 can also indirectly cause cardio-embolism and IS by causing myocardial ischemia or cardiac arrhythmias [79, 80]. It was found that people with existing cardiac diseases (coronary heart disease, heart failure, stroke), cardiovascular risk factors (e.g., age, hypertension, diabetes) and other comorbid conditions (e.g., chronic obstructive pulmonary disease, chronic renal failure, cancer) are more likely to experience more severe cases of COVID-19 and have higher mortality rates [81].

Recent research reported that after the initial 30 days of infection with COVID-19, individuals are at an increased risk of developing cardiovascular diseases, including stroke, heart rhythm issues, ischemic and non-ischemic heart disease, pericarditis, myocarditis, heart failure and coagulation issues. This risk and burden exist even for those who did not require inpatient care during the first episode and increases along with the intensity of care required. It is clear that those living through COVID-19 have an increased risk of cardiovascular issues in the year following infection. For these people, healthcare providers should include plans and treatments for cardiovascular health and treatment to address any issues that come up [82]. On the contrary, a meta-analysis findings point towards hypertension, diabetes, and cardiovascular diseases possibly being independently linked to the development of severe COVID-19, and that age and gender may be useful in predicting the risk of experiencing severe symptoms of the disease. Although it should be noted that potential confounding variables may come into play, the results do suggest that these factors might be significant [83]. Apparently, there is still a room for further investigation to examine the potential contribution of cardiovascular diseases into the severity of COVID-19.

## Significance of immune landscape in COVID-19

Immune response in SARS-CoV-2 infected patients varies, for example, asymptomatic patients are found to show weaker antiviral response [84], whereas the symptomatic patients show a swift fall in peripheral lymphocytes and a rise in neutrophils, and monocytes, causing an abnormal immune response, or immune disorders [85, 86]. It is noteworthy that some patients show relapse due to weak virus-eliminating immune responses against SARS-CoV-2 [87]. Critically ill patients develop acute respiratory distress syndrome (ARDS), and multiple organ failure because of the stronger immune response [88]. Interestingly, the body’s natural defense against viral infections involves a quick and well-coordinated immune response, which can also affect the adaptive immune response against foreign and self-antigens. Pathogenesis of severe COVID-19 pneumonia involves a dysregulated autoimmune response, causing cytokine storms leading to poor prognosis [89]. Therefore, the effect of viruses on the immune system can help us in the prevention and treatment of such a pandemic. In asymptomatic patients, i.e., patients only have qRT-PCR specific for the disease, there is a specific mild immune response which eventually leads to the rapid disappearance of the neutralizing antibodies [90]. Specific T-cell response is similar in asymptomatic and symptomatic cases with high levels of IFN-γ and IL-2 in asymptomatic patients. A highly functionalized viral-specific immune response may be triggered due to weak antiviral immunity leading to the proportional secretion of IL-10 and proinflammatory cytokines such as IL-6, IL-1β, and TNF-ɑ, occurring disproportionately in symptomatic cases [91]. Reinfection usually occurs after 4-5 months and does not always lead to new positive tests. CD4+ and CD8 + T-cells are found in the recovered patients which produce spike proteins, nucleocapsids, proteins, and membrane proteins. Antibodies produced during the previous infection are not detectable during reinfection and whether the mutation of the spike protein causes reinfection, is not certain. However, during the early stages of reinfection, high-affinity IgG and high titer antibodies produce a stronger antibody response and the absence of IgM is associated with reinfection [92–96].

Abnormal inflammation and immune response of SARS-CoV-2 infection require increased levels of proinflammatory cytokines like IL-6, IL-8, IL-1β, IL-17, G-CSF, IP-10, MCP1, MIP1ɑ, TNF, C- reactive protein and D-dimer, which produce cytokine storm, further causing local or systemic tissue damage [97]. Excess levels of dimer and cellulose cause extensive capillary coagulation reactions leading to inflammation and blood clots in the lungs, heart, kidney, nervous system, bone marrow, and vessels. The prokaryotes can infect respiratory systems, kidneys, and gastrointestinal tract in a number larger than immune cells like- macrophages, monocytes, lymphocytes (mostly CD4 + T-cells), eosinophils, and neutrophils [98]. The damage to the lungs includes hyaline membrane formation, fibrin exudate, epithelial damage, and diffuse type- II lung cell hyperplasia. Lesions in alveoli include diffuse alveolar damage, fibrin membrane, and fibrin clump formation. Moreover, inflammation and microthrombus can be found around heart capillaries, liver sinuses, and renal tubules [99, 100].

Notably, SARS-CoV-2 infection can increase the level of IL-1ɑ and IL-6 and decrease that of Th2 cells, Th17 cells, and Treg, which causes CD4 + , CD8 + T-cells, and activated CD4 + T-cells exhaustion i.e., lymphopenia. This leads to the production of CD4 + T-cells, CD8 + T-cells, B-cells, and NK-cells with the damage to CD8 + T-cells being more significant [101]. During recovery from COVID-19, the levels of Treg, activated CD4 + T-cells, and depleted CD8 + T-cells are reduced and that of B-cells is found to be increased. During the end phase of recovery, the levels of IL-1ɑ, IL-1β, IL-6, IL-10, and TNF-ɑ decreases [102]. After recovery the levels of IL-10 increases, the frequency of Th1, Th2 and Th17 cells increases, and that of B-cell decreases [103]. The level of lymphocytes signifies that in recovered patients, despite improved immunity, SARS- CoV-2 can cause long-term damage to the immune system. IL-6, IL-10, TNF, neutrophils, and dendritic cells cause lymphopenia [104]. Excessive activation of T-cells or expression of proapoptotic molecules promotes the depletion of T-cells. CD4+ and CD8 + T-cells infiltrate interstitial spaces around wider bronchioles and blood vessels. However, programmed cell death protein-1(PD-1) and PD-L1 protein are not detected on the surface of lymphocytes and no viral infection is detected in lymphocytes and mesenchymal cells. Moreover, S-protein was not found together with T-cells [105, 106].

It is believed that damage to lymphocytes by oxidative stress can initiate lymphopenia. CD4+ and CD8 + T-cells and macrophages infiltrate the interstitial myocardium without damaging myocardiocytes. Myocardial fibres undergo hypertrophy. Iron catalysed regulatory cell death occurs due to the extreme peroxidation of fatty acids. Myocarditis is lymphocytic inflammation including massive CD20 + B-cells, CD3 + T-cells, CD68+ macrophages without eosinophils, giant cells, or granulomas. Moreover, fibroblasts/macrophages promote myocardial destruction [107–109]. Despite the pathogen eradicating role of macrophages, it has also been reported to produce cytokines, enzymes, and ROS, therefore generating cytokine storms. Due to pneumonia, the alveolar cavity decreases along with the presence of many atypical and massive CD61+ megakaryocytes, neutrophils, and lymphocytes. CD68+ macrophages possess intracytoplasmic phagocytosis, eosinophilic hyaloplasm, or hemophagocytic and multinucleated giant cells. Alveolar macrophages secrete IL-6, IL-10, and TNF-ɑ. Pulmonary inflammatory macrophages produce interferon in the early stage, while continuous production of IFN-γ causes extravagant macrophage activation [110–112]. Additionally, SARS-CoV-2 viruses infect monocytes which differentiate into tissue macrophages and replicate in their cytoplasm. COVID-19-specific antibodies combine with FcR and enhance virus uptake by macrophages. ACE2 receptor expressed on the surface of lung macrophages interacts with S-protein and allows the SARS-CoV-2 virus to enter the macrophage. S-protein can also interact with ACE2 present on CD68 and CD169 macrophages in the spleen’s marginal area and marginal sinuses of lymph nodes, which upregulates IL-6 production by macrophages. Alveolar macrophages also express PD-L1 [106, 111, 113].

Therefore, it is apparent that CD169 macrophages, like Trojan horse, facilitate viral transmission, excessive inflammation, and activation-induced lymphocyte death [114]. Interestingly, MERS-CoV infects and colonizes phagocytes, replicates, and attenuates innate immunity in the host [115]. *Mycobacterium tuberculosis* & silica sand are swallowed by phagocytes, which rupture lysosomes to release hydrolase and promotes inflammation, eventually leading to the death of phagocytes. This produces severe inflammation and fibrosis like one produced by SARS-CoV-2 pneumonia [116–119]. It is noteworthy that nervous system inflammation in COVID-19 patients may or may not be because of viral infection. Severe neurological diseases show lymphocytes or mononuclear inflammatory infiltration in meningeal and cortical tissue producing neurovascular brain injury or microvascular dysfunction. CSF of patients with encephalitis, meningitis, or acute disseminated encephalomyelitis have negative RT-PCR. Only RT-PCR of CSF from patients with epilepsy was found to be positive. CSF analysis of patients with neuromuscular disease shows dissociated cytology of albumin [120–122].

SARS-CoV-2 virus releases ROS, which produce oxidative stress injury. T and B cells and NK cells encounter antigens on the virus surface, get stimulated, and later undergo apoptosis due to oxidative stress, therefore decreasing lymphocytes [102]. If infected, phagocytes capture near odour and taste cells, the virus causes oxidative stress damage to the cells, leading to odour and taste disorders. If the rupture is near the meningeal space, the virus produces oxidative stress damage to the cerebral cortex and induces brain inflammation. Nervous system of patients with epilepsy suffers severe damage because phagocytes rupture after penetrating the spinal cord tissue and invade the spinal fluid which further enters CSF.

Importantly, SARS-CoV-2 is an acidophilic anaerobic virus. According to haem theory, it suppresses haem metabolism by dissociating haemoglobin into haem and hunting porphyrin for iron. E-protein has iron-linked sites which possess catalytic properties like cytochrome C oxidase which captures iron, and generates ROS to damage the immune system, therefore producing high infectivity. If these sites had iron catalytic dismutase, catalase, and peroxidase, it could produce ROS like O-, H_2_O_2_ or OH. Peroxidase can produce hydroxyl radicals which can damage cell membrane proteins & nucleic acids. This can initiate peroxidation and rupture of lysosomal membranes [123, 124], signifying their crucial role of immune landscape in COVID-19.

## Role of redox imbalance in neurological implications of COVID-19

Many glycosylated Spike proteins cover the surface of severe acute respiratory syndrome coronavirus 2 (SARS-CoV-2), which fuses with the lung cell membrane and binds to the ACE2 in the respiratory tract [15, 17]. Apart from its role in SARS-CoV-2, ACE2 is well known for its role in hypertension. The enzyme exerts its function through cleaving angiotensin I (Ang I) or angiotensin II (Ang II) into inactive peptides Ang (1-9), and Ang (1-7) respectively. Ang (1-7) is a vasodilator and hence counteracts the vasoconstrictor effects of the ACE-Ang II axis [125]. The activity of ACE2 is vital for host entry and subsequent pathogenesis. SARS-CoV-2 infection abates the ACE2 receptor, which increases the cellular concentration of angiotensin resulting in an elevated level of oxidants, oxidative stress, and inflammation [126–128]. ACE2 deficiency has also been associated with increased NADPH oxidase 2 (Nox2) mediated oxidant formation, resulting in oxidative stress and thrombotic events in COVID-19 patients [129, 130]. Interestingly, Nox can also be activated by the release of TNF-α during the proinflammatory cytokine storm, contributing to local oxidative stress and endothelial dysfunction [131]. Moreover, in biopsies obtained from COVID-19 patients, suppression of nuclear factor erythroid2 related factor 2 (NRF2) antioxidant gene was observed. The report further showed that treatment of cells with NRF2 agonists induced a strong antiviral response, pointing to the potential role of Nrf2 in the COVID-19 management [132].

**Fig. 3:**
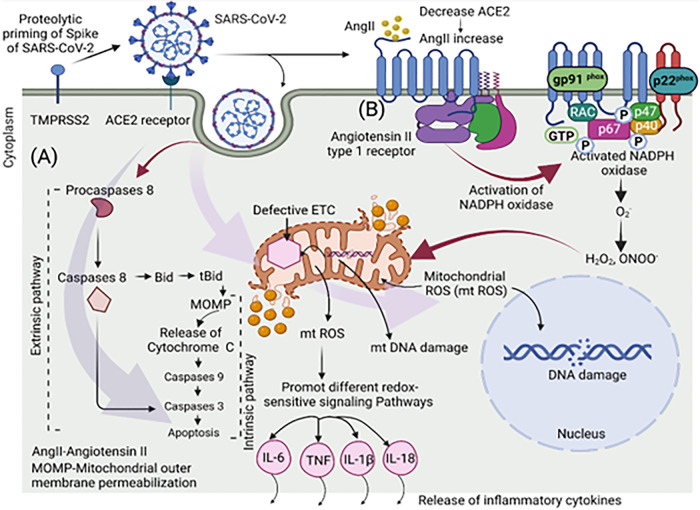
Schematic illustration of redox imbalance in the endothelial cell upon infection with SARS-CoV-2. **A** The redox imbalance in the endothelial cell occurs through AngII/AT1R/Nox axis pathway. TMPRSS2 cleaves and activates the SARS-CoV-2 spike protein (S) for membrane fusion. SARS-CoV-2 interacts with the ACE2 receptor and is taken up by the cell through endocytosis. The interaction of viral glycoproteins with ACE2 receptor leads to downregulated expression of ACE2, thereby increasing the level of angiotensin II (Ang II). The reduced ACE2 activity may facilitate Ang II binding to the type 1 angiotensin receptor (AT1R). SARS-CoV-2 causes activation of NADPH oxidase, which results in the production of reactive oxygen species (ROS). The ROS generated from the NADPH oxidase are engaged in processes that cause damage to the electron transport chain (ETC) leading to DNA damage in mitochondria as well as in the nucleus. The ROS injury to mitochondrial DNA (mtDNA) may cause mitochondrial dysfunction by impairing ETC complex formation. The mtROS also promote the activation of the different redox signaling pathways and induce the production of various inflammatory cytokines. **B** Apoptosis induction by the SARS-CoV-2 infection through both the extrinsic and the extrinsic pathways. SARS-CoV-2 may cause apoptosis by the extrinsic route, which involves the cleavage of Bid to tBid (truncated Bid) by activated caspase-8. This cleavage activates mitochondrial outer membrane permeabilization (MOMP) effector proteins BAX and BAK. The MOMP permits executioner caspases to enhance apoptosis. It also triggers the release of mitochondrial cytochrome C, which in turn triggers activation of caspase-9 and leads to apoptosis. (Figure prepared with Biorender).

The activation of AngII/AT1R/Nox axis via the generation of ROS, has been found to be associated with mitochondrial injury. The interruption of mitochondrial reduction-oxidation (redox) homeostasis, and an increase of free radicals have been linked to abnormal function of the RAS. The dysregulation of the RAS system has been linked to a series of pathological conditions due to its effect on the function of mitochondria [133]. Since AngII has been demonstrated to promote ROS generation by increasing Nox activation, it is possible that an increase in Ang II levels is one mechanism responsible for rising mtROS production and consequent endothelial cell pathologies (Fig. 3A) [134]. After NADPH oxidase generates O_2_, it reacts with NO to make ONOO^-^, which is toxic to mitochondrial respiratory complexes via tyrosine nitration and cysteine oxidation [133]. Interestingly, humans and other mammals differ in that human macrophages and other cells, when exposed to proinflammatory conditions, cannot express the NOS2 enzyme. As a result, the synthesis of NO is minimal and peroxynitrite’s potential harm cannot be factored in [135], which warrants further investigation. One of the most recognizable signs of mitochondrial dysfunction is the inability of the electron transport chain (ETC) complexes to operate properly, which leads to an increase in oxygen reduction and the consequent formation of mitochondrial reactive oxygen species (mtROS) owing to the buildup of electrons that can’t be utilized for ATP synthesis [136–140]. Mutations and DNA damage may also be caused by oxidative changes to DNA sugar-phosphate backbones, which can be induced by O_2_^-^ generating enzymes like NADPH oxidase and mitochondria. Mitochondrial dysfunction is believed to be induced by ROS damage to mtDNA, which leads to a lack of correct formation of ETC complexes [137, 141, 142]. Moreover, H_2_O_2_, a membrane-permeable mtROS, interacts with intracellular iron to form extremely reactive hydroxyl radicals which damage nuclear DNA [143–145]. SARS- CoV-2 infection also activates the intrinsic and extrinsic pathway of apoptosis (Fig. 3B) [146, 147]. It activates the caspase 8 which causes the cleavage of Bid to tBid (truncated Bid). The tBid promotes the activation of mitochondrial outer membrane permeabilization (MOMP) effector proteins BAX and BAK, which further trigger the recruitment of other caspases to perform apoptosis [148]. It also triggers the release of cytochrome C that activate caspases 9 and triggers the apoptosis [146]. Furthermore, the release of cytochrome C and p38 mitogen-activated protein kinase (MAPK)/Jun N-terminal kinase (JNK) cascade activation has been related to elevated ROS levels. Moreover, SARS-CoV-2 infection can destroy lung cells by activating a local immune response mediated by macrophages and monocytes; these cells release cytokines such as interleukin-6 (IL-6), interferon-γ (IFN-γ), and or tumor necrosis factor (TNF) into the blood, thus activating T-cells. Other inflammatory pathways activated by Ang II involve the transcriptional nuclear factor NF-kB and the expression of proinflammatory cytokines such as IL-6, IL-1β, and TNFα [149]. In COVID-19 patients, an excessive cytokine release has been documented, which induces an increase in leukocyte recruitment to different body organs leading to multiorgan failure, a phenomenon called cytokine storm syndrome [150]. It is crucial to understand that oxidative stress and inflammation are mutually augmenting each other [151]. Persistent oxidative stress and lipid peroxidation correlated with inflammasome activation, and both cooperatively contributed to disease severity [152].

Overwhelming production of ROS results in local or systemic tissue damage leading to severe COVID-19 infection. Oxidative stress is also known to increase the formation of neutrophil extracellular traps (NETs) and suppress the adaptive immune system [153]. NETosis is the cell death of neutrophils where neutrophils extrude DNA and antimicrobial proteins in the extracellular space, forming a net-like structure that traps and kills invading pathogens [154–156]. Elevated release of NETs was reported to occur in severe cases of COVID-19 [157]. Sera from COVID-19 patients revealed increased levels of extracellular DNA and MPO-DNA and citrullinated histone H3, specific markers of NET. The levels of extracellular DNA showed a strong correlation with other markers of inflammation (e.g., C-reactive protein, D-dimer). The levels of both extracellular DNA and MPO-DNA were much higher in patients on mechanical ventilation compared to regular COVID-19 patients. The study interestingly showed that sera from COVID-19 patients triggered NET release from control neutrophils in vitro, indicating that circulating NETs can potentially be used to predict the extent of disease severity. The excessive accumulation of NETs causes damage to the host by inducing proinflammatory mechanisms [158]. NETs instigated immune thrombosis in COVID-19 patients and therefore were suggested as a suitable therapeutic target to mitigate prothrombotic complications in COVID-19 patients [159]. The interaction of SARS-CoV-2 with hemoglobin results in dysregulated iron metabolism and iron-mediated oxidative stress and hyperinflammation. Ferroptosis-regulated cell death through iron is initiated by iron and oxidant-induced lipid peroxidation [160]. Oxidized phospholipid thus in a vicious cycle exacerbates NET formation [161–163].

Mitochondria are the primary sources of ROS production in cells; therefore, mitochondrial dysfunction can potentially play a major role in the oxidative stress observed in SARS-CoV-2 viral infection. Recent studies have hypothesized that the cytosolic ROS produced by Nox could trigger the opening of the ATP-sensitive mitochondrial potassium channel, causing the depolarization of the mitochondrial membrane and a burst of the mitochondrial ROS production [164]. Inflammatory response and immune signals trigger intracellular cascades that alter the mitochondrial metabolism [165]. For example, TNF-α and interleukin (IL)-6 impede mitochondrial oxidative phosphorylation and couple ATP production and trigger the production of mitochondrial ROS in the cell. This leads to mitochondrial dysfunction, which has been found in patients with COVID-19 [166]. The mitochondria-targeted antioxidant SkQ1 (10-(6’-methylplastoquinonyl) Decyltriphenylphosphonium) inhibited ROS production induced by the chemo-attractant fMLP via G-protein coupled receptor. It was concluded that mitochondrial ROS are involved in assembling and activating the multicomponent enzyme complex Nox2, which is the main source of ROS in the neutrophils [167].

Since ROS play a key role in neutrophil activation, it is thus a plausible approach to target agents that could inhibit the damaging activity of neutrophils and reduce their number in inflammatory lesions. Oxidative stress is implicated in the pathophysiology of all factors causing COVID-19 and post-COVID-19 symptoms. ROS triggers inflammation, damages the endothelium, promotes the formation of autoantibodies, and disrupts neurotransmitter assembly. Oxidative stress presumably is an important cause of post-COVID-19 symptoms and thus a driving force in an immuno-endothelial-neurological vicious circle [168].

## Application of bioinformatics in the study of neurological implications of COVID-19

Bioinformatics analysis has already played an essential role in multiple aspects of COVID-19 research [169], including genomic epidemiology [170–172], host-pathogen interaction [173–176], vaccine development [177–179], and prognosis [180, 181]. Recently, researchers recognized bioinformatics tools as a highly efficient approach to deriving novel insights into the neurological implication of COVID-19. Sepehrinezhad et al. showcased a computational pipeline of drug repurposing with publicly available databases and online tools [182]. They collected gene sets related to COVID-19 and neurological disorder, respectively from GeneWeaver [183]. Combining the 139 shared genes with the functional interaction information from String, they used Cytoscape to construct gene-gene interaction networks and identified a few central genes, such as IL-6, TNF, and AKT, closely related to the neurological manifestation of COVID-19. WebGestalt [184] results showed that polaprezinc, andrographolide, and etanercept can be repurposed to target those genes playing central roles in COVD-19 and neurological disorders. In another study with a similar approach, Rahman et al. [185] studied the interaction between COVID-19 and neurological diseases. They identified related gene sets through differential expression analysis of the transcriptomic profiles of COVID-19 patients and neurological disease patients, respectively. Downstream analysis of the shared differentially expressed genes (DEG) included GO enrichment analysis and interactome analysis, which were like Sepehrinezhad et al.’s work. The difference is that Rahman et al. emphasized the similarity between COVID-19 and neurological diseases. They identified a substantial overlap of DEGs and GO terms between COVID-19 and five neurological diseases, including Parkinson’s disease, Alzheimer’s disease, Amyotrophic lateral sclerosis, Huntington’s disease, and multiple sclerosis [185].

In another research, a more network-based approach has been adopted [186]. From a list of 332 genes known to interact with COVID-19, the authors selected the target genes and their neighbors from the brain-specific interactome from TissueNet [187]. The resulting subnetwork was named the COVID-19 target network (CTN). Cytoscape was used to identify functional modules and key genes in each module. The spatial-temporal expression analysis of the key genes in each module through BEST (Brain Expression Spatial-Temporal) indicated COVID-19 may induce impairment of sensory systems, memory, and cognition. Drug repurposing analysis of the key genes and genes bridging functional modules identified many FDA-approved drugs, such as fulvestrant, tamoxifen, and calcitriol. Prasad et al. have also investigated the comorbidity of COVID-19 with other brain diseases by constructing disease-disease interaction networks with the Gene ORGANizer database [188]. The resulting network showed that several diseases like ataxia, dysarthria, spasticity, cerebral atrophy, autism, dementia, and stroke were closely related to COVID-19 and are also connected with multiple genes in the brain. In addition to neurological disorders in general, computational researchers have investigated the interaction between COVID-19 and specific neurological diseases. Cen et al. investigated COVID-19-related stroke by identifying hub genes and functional modules in a weighted gene co-expression network [189]. Wang et al. studied how COVID-19 affected Alzheimer’s disease by integrating transcriptomics, epigenomics, and proteomics to perform enrichment analysis and network analysis [190].

Current bioinformatics applications to the neurological manifestations of COVID-19 mainly focused on applying enrichment analysis and network analysis to derive novel biological insights from public data sources. Shared genotypes between COVID-19 and neurological diseases and related targeting drugs have been identified. However, the results across studies are not consistent. Shared genes and suggested drugs are largely different across bioinformatics studies. It may be due to different computational pipelines and different databases. Future biological research is warranted to validate these computational results.

## Conclusions and outlook

The emergence of severe COVID-19 cases has led to an increase in neurological complications. Such symptoms include anosmia, agnosia, stroke, paralysis, cranial nerve deficits, delirium, encephalopathy, meningitis, and seizures. Research suggests that such neurological effects of the novel coronavirus SARS-CoV-2 are aided by its entry and binding into the CNS through ACE2, NRP1, TMPRSS2 and CD147, receptors. In addition, COVID-19 can also trigger an autoimmune response that damages the nervous system. This can lead to long-term neurological problems, such as fatigue, cognitive impairment, and pain.

The immune landscape in COVID-19 is also important to consider. The virus can evade the immune system by mutating its surface proteins. This makes it difficult for the body to mount an effective response and can lead to more severe disease. In addition, the immune response itself can be harmful, as it can damage the nervous system. Redox imbalance is another factor that may contribute to the neurological complications of COVID-19. Redox imbalance is a condition in which there is an excess of free radicals in the body. Free radicals are unstable molecules that can damage cells and tissues. COVID-19 can lead to redox imbalance, which may contribute to the development of neurological symptoms. Bioinformatics is a rapidly growing field that is being used to study the neurological complications of COVID-19. Bioinformatics can be used to analyse large datasets of patient data, which can help to identify new risk factors and treatment targets. Bioinformatics can also be used to develop new models of the disease, which can help to improve our understanding of how COVID-19 affects the nervous system.

To generate a more comprehensive field of knowledge surrounding the neurological implications of COVID-19, examination of blood and cerebrospinal fluid samples for suggestive markers of inflammation, both systemically and in the CNS, is necessary. In addition, postmortem neuropathological studies of patients who have lost their lives to COVID-19 are needed to assess the potential neurological effects of the virus. Baseline neurological assessments and clinical history recordings of those who have recovered from the virus should also be executed to evaluate their potential exposure to prolonged dormant SARS-CoV-2 and its possible risk of leading to long-term neurological consequences. It is essential that further studies be conducted to craft effective strategies that will help lessen the severity of the disease and ultimately improve the treatment outcomes of those with pre-existing neurological conditions.
